# Supplementary material for: Genome Mining Uncovers NRPS and PKS Clusters in Rothia dentocariosa with Inhibitory Activity against Neisseria Species
Source: Antibiotics (Basel). 2023 Nov 4;12(11):1592. doi: 10.3390/antibiotics12111592 (PMC10668837; doi:10.3390/antibiotics12111592)
Supplement: Supplementary file 1 [file antibiotics-12-01592-s001.zip › antibiotics-2685892-supplementary.pdf]

(a) Enterobactin non-ribosomal peptide synthetase EntF

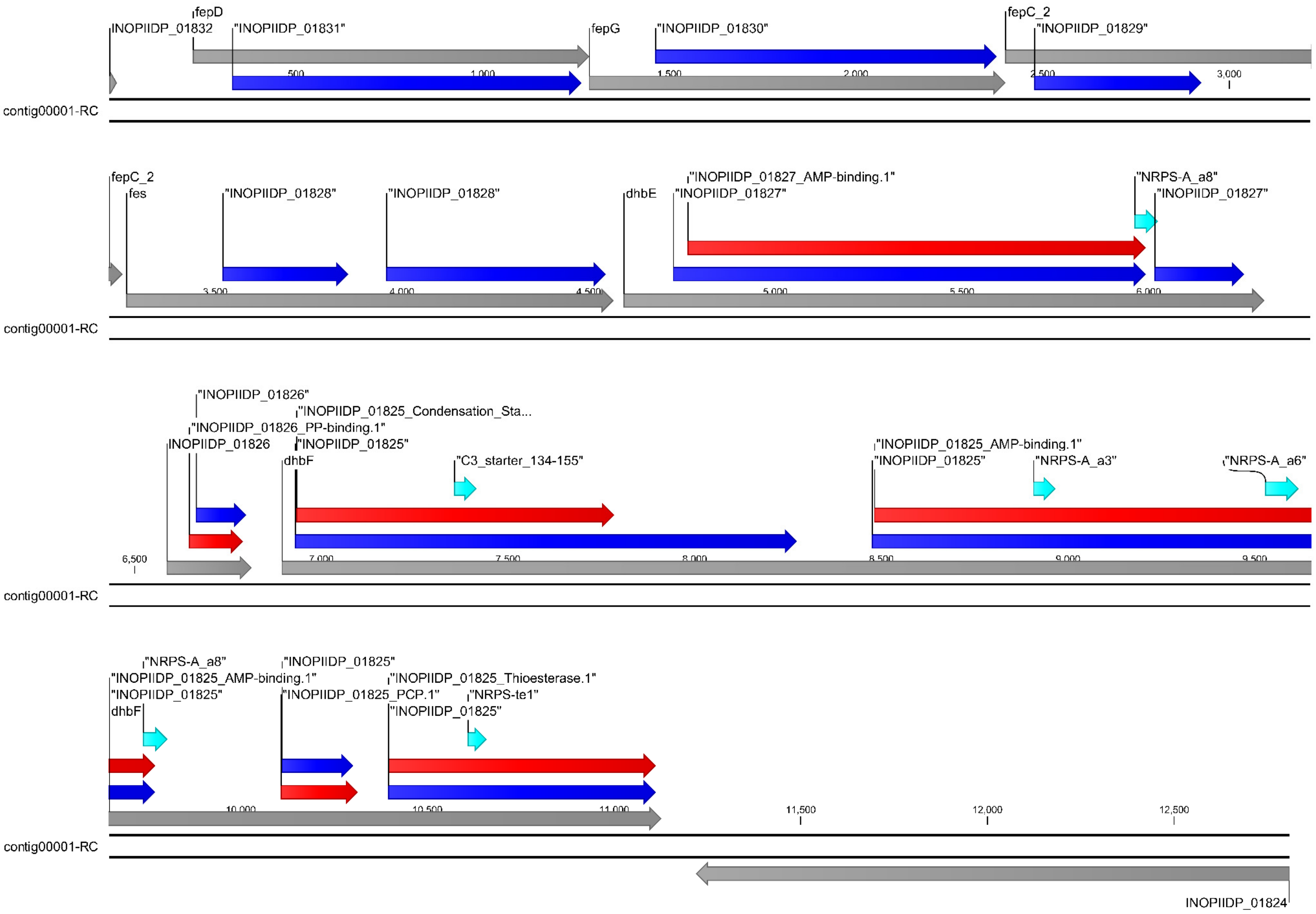

(b) Actinorhodin polyketide putative beta-ketoacyl synthase 1 and 2

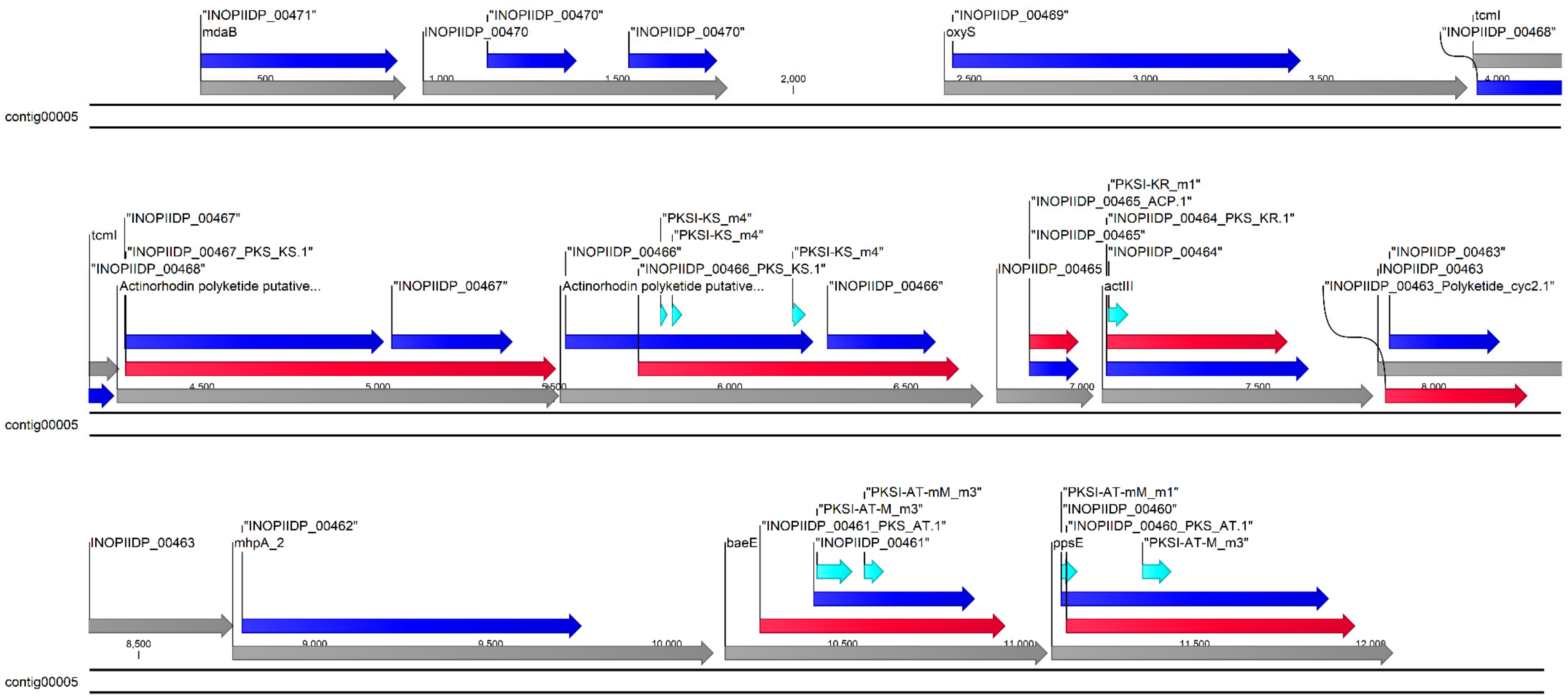

(c) Type 2 lanthipeptide synthetase LanM

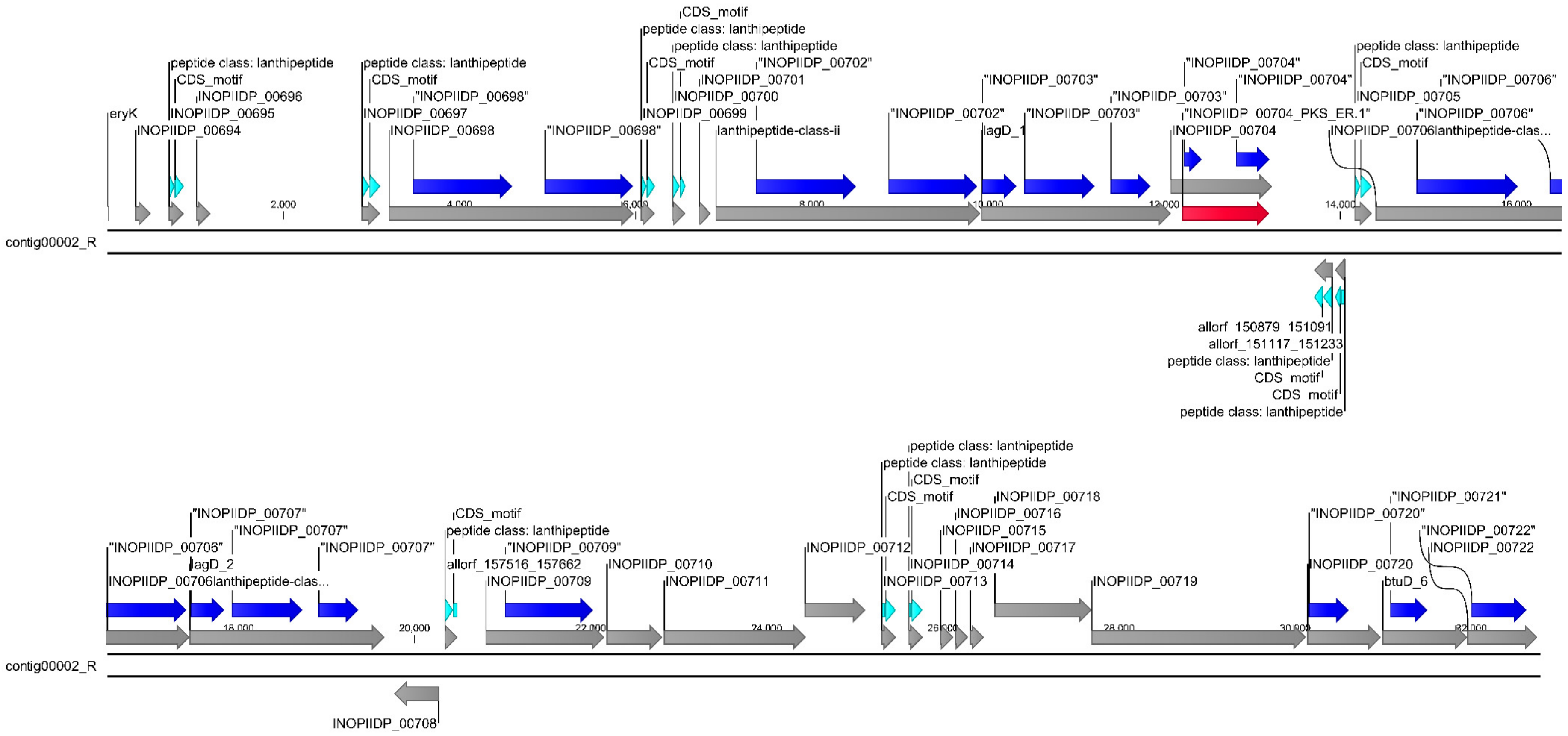

(d) YcaO-like family protein and SagB family peptide dehydrogenase (LAP)

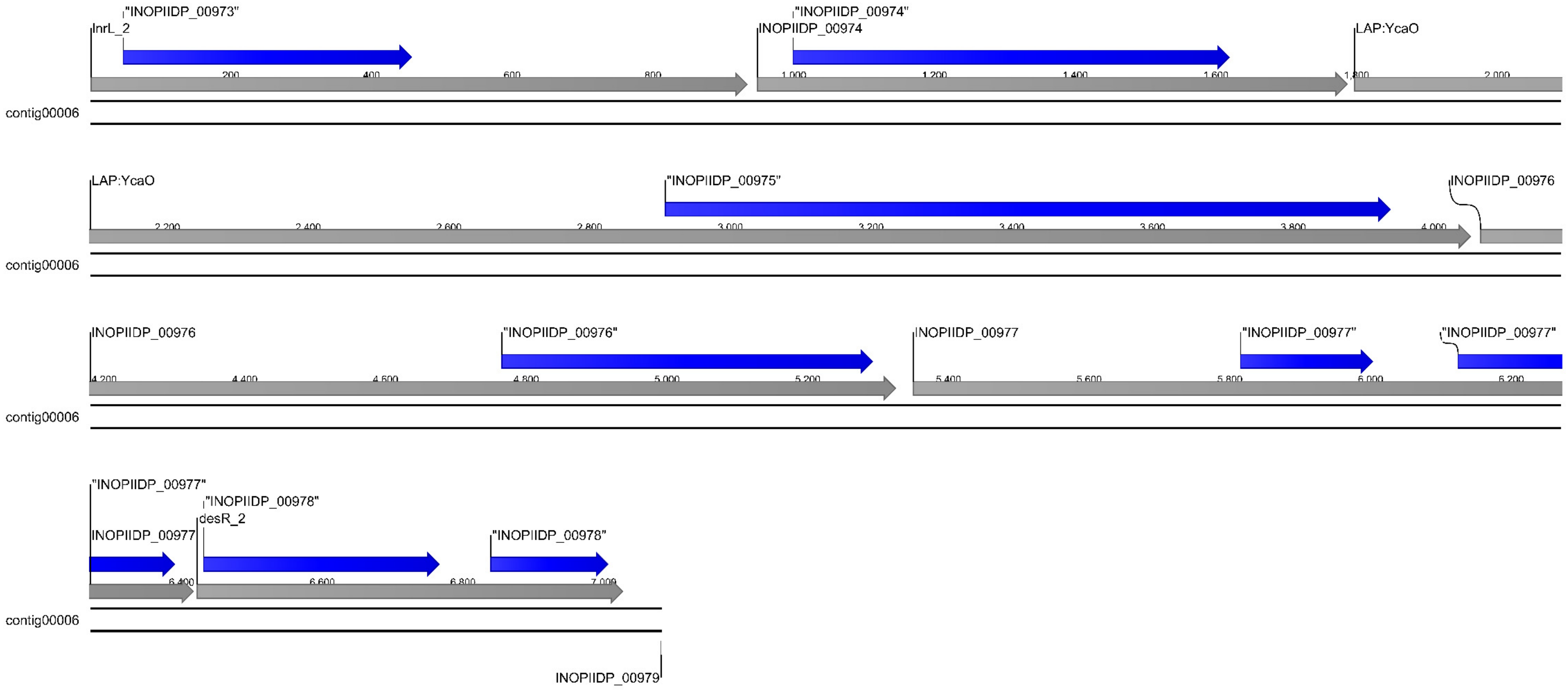

(e) Butyrolactone

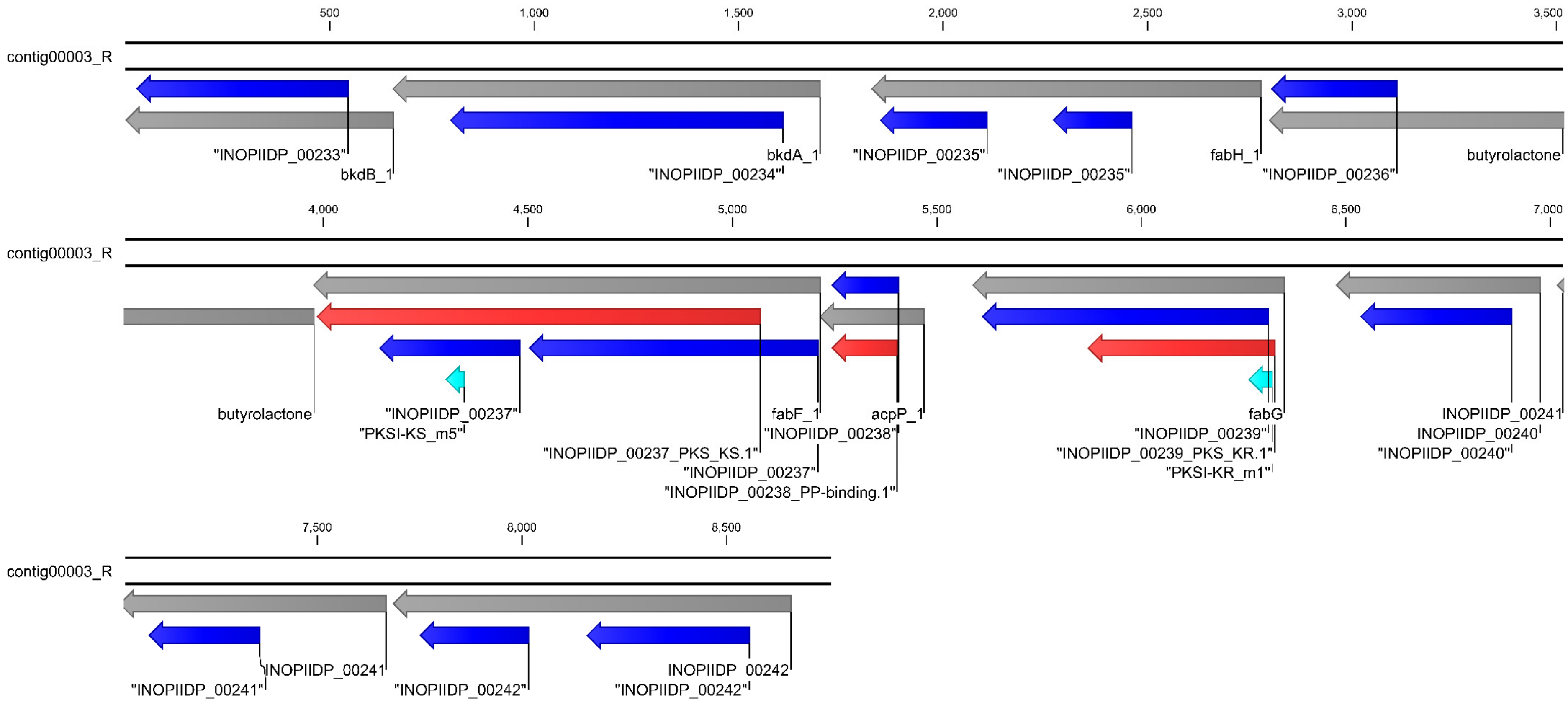

Supplementary Figure S1: The putative genetic organization of (a) enterobactin, EntF (b) actinorhodin polyketide putative beta-ketoacyl synthase 1 and 2 (c) lantipeptide-class-ii peptide (d) LAP (Linear azol(in)e-containing peptides) (e) Butyrolactone.

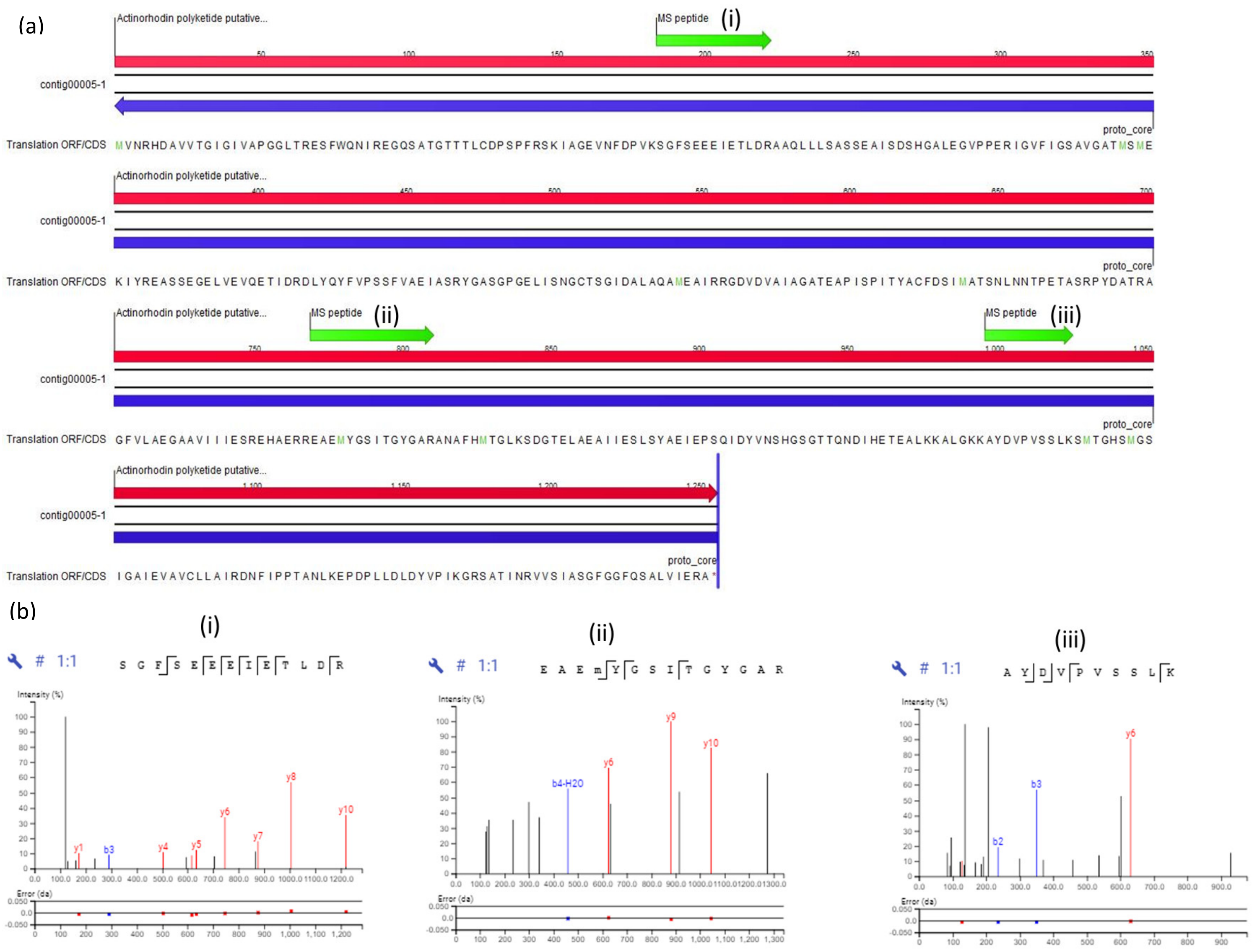

Supplementary Figure S2: Putative genetic organization of (a) enterobactin, EntF and (b) corresponding three peptides identified using LC-MS system.
